# Chinese science education in schools and beyond

**DOI:** 10.1093/nsr/nwz017

**Published:** 2019-03-07

**Authors:** Zhonghe Zhou

**Affiliations:** 1 Institute of Vertebrate Paleontology and Paleoanthropology, CAS, China; 2 Associate Editor-in-Chief of NSR

The latest national survey announced by the China Association for Science and Technology indicates that science literacy for adults was around 8.5% in 2018—a significant leap over the last 3 years, yet still low compared to developed countries. Further increase in science literacy is critical for realizing China's ambition of becoming a strong country in science and technology. One of the necessary moves that should be taken is to improve the now inadequate science education not only in primary and high schools, but also beyond schools, namely continuing science education for adults.

For historical reasons, science education has long been outdated or inadequate in Chinese schools, often with more emphasis on mastering existing knowledge rather than cultivating interest and skills of problem solving, interpretation and inferencing. Progress has been happening in China since 2017—that is, science is now taught from Grade 1 in primary school as a separate subject nationwide, and new textbooks and curricula for science education are under revision based on opinions from scientists. However, Chinese schools also suffer large class sizes and a lack of qualified science teachers, and more efforts are needed to improve the efficiency of science education in schools, including the reform of the current entrance-examination system that has given students and teachers as well parents too much pressure to be involved in learning science.

In addition to science education in schools, continuing adult science education is also a challenging issue for China today, given the importance of science literacy to increasing the influence of public interest and engagement in societal affairs such as food safety, health care, the environment, legal issues, as well as ethical concerns resulting from the rapid progress in biotechnology, artificial intelligence, etc. Adult science education is also critical for securing jobs and professional advancement as well as learning the necessary skills to adapt to the society that is changing at an unprecedented rate in China and the rest of the world.

For government employee and officials, particularly policymakers, the need for continuing science education may be more urgent in China today, considering the fact that science and technology are playing an increasingly important role for its social and economical development. It is also notable that, since some of the most complicated and urgent public-policy debates in China and elsewhere are, at their center, issues related to science, it is critically important for policymakers to be well informed, with an updated and authentic scientific background about the pros and cons of important public-policy decisions.

The recent investigation of the Quanjian Group that touted many miraculous products shows the government's determination for fighting not only crime and corruption, but also the vulnerability of citizens with low science literacy to misleading commercials. Most citizens do not have direct access to factual information and are often incapable of separating the facts from propaganda. They therefore are likely to be predominantly influenced by the prevailing perception from the internet or in their community.

It must be noted that there is still a long way to go in making science education in schools at the pace of China's march towards an innovative country that is strong in science and technology. Understanding the importance of science for societal development, and cultivating an interest in science and the ability for critical thinking and problem solving through science education will offer students the skills and capability that they need to succeed in schools and beyond.

As the pace of scientific research accelerates, the government should invest more in the public engagement of science and support more formal or informal adult science education programs of various forms. And the scientific community should work with the government to help the public and policymakers to improve the capability of distinguishing science from fiction, and facts with evidence from pure speculation and claims, and focus on creating a society in which well-educated adults are equipped with scientific thinking and sufficient scientific skills to develop informed opinions about important issues that may affect society and their daily lives as well.

## Notes

Zhonghe Zhou Institute of Vertebrate Paleontology and Paleoanthropology, CAS, China Associate Editor-in-Chief of NSR

